# The relationship between self-esteem and social avoidance among university students: chain mediating effects of resilience and social distress

**DOI:** 10.1186/s40359-025-02444-2

**Published:** 2025-02-11

**Authors:** Aili Shang, Liping Feng, Guoli Yan, Ling Sun

**Affiliations:** 1 Ningxia Vocational Technical College of Industry and Commerce, No. 531, Dalian West Road, Xixia District, Yinchuan, Ningxia China; 2 Department of Clinical Psychology, Qingdao Municipal Hospital, No.1, Jiaozhou Road, Shibei District, Qingdao, Shandong 266011 China; 3 Department of Child and Adolescent Psychology, Tianjin Anding Hospital, No.13, Liu Lin Road, Hexi District, Tianjin, 300222 China

**Keywords:** Social anxiety, Social distress, Social avoidance, Self-esteem, Resilience, Chain mediation effect

## Abstract

**Backgroud:**

This study aims to explore the relationships between self-esteem, resilience, social distress, and social avoidance among college students. It also examines the mediating roles of resilience and social distress in the relationship between self-esteem and social avoidance.

**Methods:**

A convenience cluster sampling method was used to select all first-year students from a university in Yinchuan, Ningxia. Data were collected through an online survey administered via the WENJUANXING platform, which included a general information questionnaire, the Self-Esteem Scale, the Psychological Resilience Scale, and the Social Avoidance and Distress Scale. A total of 2513 first-year students completed the survey. SPSS 26.0 software was used to analyze the correlation between self-esteem, resilience, social distress and social avoidance, and the mediation model was tested by Mplus8.

**Results:**

Self-esteem, resilience were negatively correlated with social distress scores (*r* = -0.411, *p* < 0.01; *r* =-0.387, *p* < 0.01, respectively). Self-esteem and resilience were negatively correlated with social avoidance scores (*r* = -0.437, *p* < 0.01; *r* = -0.379, *p* < 0.01, respectively). Social distress and social avoidance scores were positively correlated (*r* = 0.778, *p* < 0.01). Resilience partially mediated the association between self-esteem and social avoidance(β = -0.02, *p* < 0.01), with a mediation rate of 5.01%. Social distress partially mediated the associations between self-esteem and social avoidance(β = -0.203, *p* < 0.01) with a mediation rate of 50.87%. Resilience and social distress together (β = -0.06, *p* < 0.01) formed a mediating chain between self-esteem and social avoidance, with a mediation rate of 15.03%.

**Conclusions:**

Self-esteem was negatively associated with social avoidance. Resilience and social distress were found to mediate the association partially.

## Background

Social Anxiety (SA) is one of the most prevalent forms of anxiety and a common psychological experience in social situations [1]. Social avoidance and social distress are the main behavioural manifestations and emotional responses of social anxiety problems; social avoidance is the tendency to avoid social interaction, and social distress refers to the distressing feeling of being in a social situation [2]. A theory suggests that social avoidance is a means of regulating negative emotions by reducing the likelihood of a negative outcome or reducing the expected distress associated with the social context [3, 4]. For example, people who suffer from social anxiety feel anxious before social events. In an attempt to manage their discomfort, they often choose to stay at home to avoid social situations. The subsequent sense of relief maintains the use of social avoidance by reinforcing beliefs about potential negative outcomes and emphasising the comfort associated with the feeling of staying at home to maintain the use of social avoidance. Although social avoidance can be a viable strategy in some situations, many social situations are unavoidable, even for people with social anxiety. At university, individuals experience rapid physical and mental growth, with interpersonal interactions increasingly becoming an important part of their academic lives. Social interactions during this period are crucial for the healthy development of college students’ personalities, and harmonious relationships are a key factor in promoting mental health. The interpersonal relationships at universities have become more diverse and complex, causing some students to struggle with social situations and avoid social interactions, thereby reducing their opportunities to learn and adapt to work and studies [5]. Some increase risks of internet and drug addiction [6–8].Anxiety, shyness and avoidance behaviours in social situations have increased significantly among Chinese university students in recent years, according to a study [9]. This may be related to different cultural norms in various countries, as a study [10] found that Asians are more able to tolerate to the impact of social anxiety. In China, collectivism is more prominent, with those who engage in social avoidance being less accepted by those around them. So social avoidance individuals may face more psychological pressure and should be given greater attention.

Sociometer theory suggests that self-esteem is a predictive factor of social anxiety. Self-esteem is a relatively quick automatic indicator of whether one feels accepted or rejected by others, monitoring interpersonal relationships and motivating individuals to take certain actions to minimum the possibility of being rejected or refused [11]. Low self-esteem increases sensitivity to interpersonal rejection, weakening an individual’s ability to adapt to social environments and thereby exacerbating levels of social anxiety. Yousaf’s research [12] suggested that students with lower self-esteem tend to engage in more avoidance behaviors towards social interactions, and the same is true for Wang’s study [13] in China. Han [14] believed that self-esteem can directly predict social anxiety levels, which have a significant negative correlation. He thought that social anxiety is merely an external manifestation of mental health issues among college students, with individual self-esteem being an internal factor. Therefore, understanding and cultivating protective factors between them may reduce the association between low self-esteem and social avoidance.

From a positive psychology perspective, resilience has the potential to become a protective factor in reducing the negative relationship between low self-esteem and social avoidance among students. Resilience refers to an individual’s ability to adapt positively after experiencing adversity and setbacks, demonstrating a strong capacity to rebound from difficult experiences [15].Resilience is a psychological trait with positive tendencies that enables individuals to cope effectively with stressful situations [16].Researches have shown that resilience has a protective effect on the physical and mental state when experiencing or facing adversity [17–19]. Previous research confirmed that individuals with higher levels of resilience could actively mobilize psychological resources and be more confident in interpersonal interactions, leading to good self-experiences and less anxiety [20]. In contrast, individuals with lower levels of resilience are sensitive toward interpersonal relationships, vulnerable to negative social events such as peer rejection and isolation, and prone to social avoidance, which then lead to interpersonal tension and negative emotions such as social anxiety [21]. Chen’s study confirmed that individuals with high resilience had lower levels of social anxiety [22].Self-esteem is recognized as having a central influence on resilience, helping to reduce individual vulnerability or increase individual adaptability, and is a key intrinsic factor in resilience [23]. Pakistan believes that self-esteem is significantly and positively related to resilience [24]. A survey based on artificial neural networks in university students found a positive correlation between self-esteem and resilience [25]. Positive self-esteem can be regarded as a fundamental characteristic of mental health, enabling the mobilization of surrounding resources, better adaptation to life, and acting as a protective factor in the realms of health and social behavior [26]. According to the cognitive-behavioural theory of social anxiety [27], individuals’ negative perceptions can lead to increasing levels of social anxiety. This means that people with lower self-esteem tend to have lower values, exhibit more obvious negative cognition when facing stress, have poor adaptability, and are more prone to psychological issues. If psychological resilience is enhanced through certain measures, it can boost confidence, provide positive psychological cues and cognitive approaches to overcoming difficulties, and help individuals adjust their emotions and attitudes more quickly and rationally when facing conflicts, misunderstandings, or other interpersonal challenges. This positive mindset helps reduce the negative impact of interpersonal distress on individuals. Pu [28] discovered a mediating effect of resilience on the relationship between life events and social anxiety in college students.

As mentioned above, individuals with social distress have a pattern of withdrawing from social relationships. People with high levels of social distress experience discomfort in social situations and prefer to be alone [29, 30]. One possible explanation for avoidance is the level of social anxiety. This means that a person with high levels of social anxiety may avoid or try to avoid situations that they are anxious about. In fact, moderate to high correlations between social distress and avoidance have been reported in non-clinical samples(0.68-0.76) [31]. A number of studies have been carried out nationally and internationally on the relationship between self-esteem and social anxiety, and the results have shown that there is a significant negative correlation between self-esteem and levels of social anxiety [32–36], meaning that people with low self-esteem often lack confidence and are unable to cope effectively with the stress of social situations, leading to high levels of social distress and avoidance. Therefore, alleviating social distress may reduce social avoidance in people with low self-esteem, but this has not been extensively investigated.

While the mental health challenges stemming from social avoidance have received substantial attention in research, there is a scarcity of studies in China that investigate the potential mediation effects of resilience and social distress in the associations between self-esteem and avoidance among college students. As an intrinsic psychological factor, low self-esteem is associated with higher social distress/avoidance [32]. Research has shown that resilience is a protective ability that increases adaptability and reduces social distress/avoidance [37]. In China, resilience has been shown to moderate the association between self-esteem and negative feeling. In a survey of type 2 diabetes patients, it was found that self-esteem can reduce shame by enhancing resilience [38], with resilience playing a mediating role in the relationship between self-esteem and shame. Can resilience, which can positively mobilize psychological resources and assist in emotional regulation to overcome adversity, help students with low self-esteem to improve their negative perceptions and coping styles, cope positively with social situations, and reduce social distress/avoidance? Although Sumter [31] demonstrated that social distress is positively related to social avoidance, the moderating role of resilience and social distress in the relationship between self-esteem and avoidance has gone largely unexamined.

## The current study

Although there are considerable researches on the relationships between self-esteem, resilience, social anxiety and avoidance among college students, the complex mechanisms underlying the interactions between these four factors remain unclear. Therefore, this study aims to contribute to the understanding of the complex relational mechanisms among these variables. Based on the above, it hypothesizes that: [1]self-esteem is a negative predictive factor for social avoidance in college students; [2]Self-esteem may influence social avoidance through the mediating role of resilience; [3]social distress mediates the relationship between self-esteem and social avoidance; [4]resilience and social distress act as chain mediators between self-esteem and social avoidance; with the goal of identifying effective intervention strategies for college students’ social avoidance.

## Methods

### Participants

This study was conducted from September to October 2022 using a convenient cluster sampling method. Before the investigation, we consulted with school psychology teachers to exclude students who had a history of mental disorders such as depression, anxiety, obsessive-compulsive disorder, and schizophrenia. A total of 2,713 first-year students were selected from a university in Ningxia. Among them, 2,513 students agreed to participate in the survey and completed the corresponding questionnaire, resulting in a response rate of 92.63%. The age of the participants ranged from 15 to 25 years (mean age = 19.48 ± 1.206 years). The participants included 1,331 males (52.96%) and 1,182 females (47.04%), with 1,310 of Han ethnicity (52.13%) and 1,188 of Hui ethnicity (47.27%), and 15 of others(0.6%). 1093(43.49%) of students live in urban areas and 1420(56.51%) in rural areas. 1768(70.35%) of students reported feelings of family socioeconomic status(SES), while 659 (31.84%) students had no feelings of family SES. Regarding family composition, 363(14.44%) of the students were identified as only children. Regarding parental marriage, 20% of the students’ parents remarried and 10% were lone parents. Furthermore, regarding the educational levels of parents, 2016(80.22%) of students’ fathers had not completed middle school, while 1316(52.37%) of mothers had only attained a primary school education. Lastly, in the COVID-19 epidemic setting, the highest percentage of students reported being somewhat worried about the situation (1090 (43.37%)), while 665 (26.46%) students reported being little worried and 758 (30.16%) students reported being more worried.

### Instruments

#### Demographic questionnaire

This questionnaire included questions on gender, age, ethnicity, only child status, family residence location, feelings of family SES, parental marital status, parental education level, and worries about COVID-19.

#### Social avoidance and distress scale (SAD)

The SAD scale was used to assess participants’ social avoidance and distress. It was developed by Watson and Friend in 1969, with a Chinese version revised by Ma Hong. The scale consists of 28 items, using a “Yes = 1, No = 0” scoring system, with a total score ranging from 0 to 28 points. The scale includes two subscales: social avoidance and social distress. The average item-total correlation coefficient was 0.77, with reliability coefficients for avoidance and distress subscales of 0.85 and 0.87, respectively, and the test-retest reliability coefficient was 0.68 [39]. The Cronbach’s α is 0.871 for the overall scale of SAD in this study, 0.773 for the distress subscale, 0.760 for the avoidance subscale.

#### Rosenberg self-esteem scale (RSES)

The Chinese version of the Rosenberg Self-Esteem Scale (RSES) was used to measure individuals’ self-esteem evaluation [40].This scale is widely used in Chinese population. It consists of 10 items, with items 3, 5, 8, 9, and 10 reverse scored. A 4-point Likert scale was applied, ranging from 1 (strongly disagree) to 4 (strongly agree). The total score is obtained by summing the scores of all 10 items, with higher scores reflecting higher levels of self-esteem. The Chinese version of RSES is a valid and reliable instrument to assess self-esteem among college students in China with a Cronbach’s alpha of 0.88 [41]. In this study, the Cronbach’s alpha coefficient of the scale is 0.841 and the exploratory factor analysis shows a KMO = 0.864 and Bartlett’s test *P* < 0.05, indicating good structural validity.

### Connor-Davidson resilience scale (CD-RISC)

The Connor-Davidson Resilience Scale (CD-RISC) was used to assess participants’ psychological resilience. The Chinese version, revised by Xiao Nan and Zhang Jianxin, consists of 25 items across three factors: tenacity, strength, and optimism [42].The scale uses a 5-point Likert scale (0–4 points), with higher scores indicating higher levels of resilience. The internal consistency coefficient for the Chinese version was 0.91, and the Cronbach’s α for each subscale was 0.88 for Tenacity, 0.80 for Strength, and 0.60 for Optimism [43]. In this study, the Cronbach’s alpha coefficient of the overall scale is 0.971. And the Cronbach’s α is 0.959 for the tenacity dimension, 0.925 for the strength dimension, and 0.758 for the optimism dimension. Exploratory factor analysis shows that KMO = 0.981, Bartlett tested *P* < 0.05, indicating good structural validity.

## Procedure

The specific survey procedure was as follows: First, university counsellors received training from the research team on the content of the questionnaire and ethical considerations. The counsellors were then responsible for distributing the survey, which was done by sending students a link to the questionnaire and an informed consent form via the Wenjuanxing platform. After providing informed consent, students were instructed to complete the questionnaire independently. The electronic platform ensured that all questions were mandatory and set specific value ranges for response options. Students could only submit the survey after confirming that their answers were correct. All participants in the study signed the informed consent form. This study was approved by the Ethics Committee of Tianjin Anding Hospital (2022-02).

### Statistical analysis

Statistical analyses were performed using SPSS version 26.0 and Mplus8. Initially, the demographic characteristics of college students were identified. T tests or ANOVA were applied to examine the influence of multiple socio-demographic factors on social avoidance. Subsequently, pearson correlation analysis was used to examine the relationships between social avoidance and distress, self-esteem, and resilience among students. Then hierarchical regression analysis investigated associations between self-esteem and social avoidance, and the potential mediating role of resilience or social distress in these associations was explored. Covariates, including gender, residence, ethnic, only child, feelings of family SES, parental marital status, parental education level, and worries about COVID-19, were integrated into respective models. The Model 1 only kept the demographic information. The self-esteem was added into Model 2, and resilience was added into Model 3, while social distress was added into Model 4. Statistical significance was set at 0.05 using a two-tailed test. Using self-esteem as the independent variable, social avoidance as the dependent variable, and resilience and social distress as the mediator variables, the structural equation model and a chained mediation effect test was constructed using Mplus 8.0. Upon confirming the assumed relationships, statistical significance testing and path coefficient calculations were executed via 5000 resampling bootstraps. A mediation effect was considered statistically significant if the confidence interval did not include zero.

## Results

In this study, the questionnaire method was used, and in order to avoid common method bias, the Harman single-factor test was used to test it [44]. The results showed that a total of 63 factors were analysed for all survey items without rotation, and the mutation rate interpretation of the first factor was 27.775%, which was less than the critical value of 40%, indicating that there was no obvious deviation of the common method in this study.

Table 1 displays the associations between social distress, social avoidance, social anxiety and student demographic characteristics. The scores of social distress, social avoidance and social anxiety in college students varied widely by gender, all showing that females scored statistically significantly higher than males, T scores are − 7.888(*P* < 0.01), -7.583(*P* < 0.01), -8.210(*P* < 0.01), respectively. Students’ feelings of family SES showed increased levels of social distress, social avoidance and social anxiety (T = 4.425, *p* < 0.01; T = 4.281, *p* < 0.01; T = 4.597, *p* < 0.01). Higher mothers’ education (Middle school or above) was associated with lower levels of social distress and social anxiety (T = 2.947, *p* < 0.01; T = 2.543, *p* < 0.05). Students more worried about the COVID-19 epidemic had higher social distress than those little or somewhat worried(F = 3.206, *p* < 0.05). In contrast, on the scores of social distress, social avoidance and social anxiety, there were no statistically significant differences for ethnicity, living in urban or rural, whether they were an only child or not, their father’s education.

**Table 1:** The differences of Social Distress/Avoidance($\bar X$ ± s) among demographic information

Variable	Group(*n*)	Distress	T/F	*P*	Avoidance	T/F	*P*	Social anxiety	T/F	*P*
Gender	Male(1331)	6.785 ± 3.428	-7.888^**^	0.000	6.263 ± 3.295	-7.583^**^	0.000	13.048 ± 6.323	-8.210^**^	0.000
	Female(1182)	7.890 ± 3.571			7.277 ± 3.404			15.168 ± 6.577		
Residence	Urban (1093)	7.345 ± 3.801	0.491	0.623	6.850 ± 3.636	1.402	0.161	14.195 ± 7.050	0.992	0.321
	Rural (1420)	7.274 ± 3.324			6.655 ± 3.176			13.930 ± 6.097		
Ethnic	Han(1310)	7.321 ± 3.656	0.063	0.939	6.754 ± 3.536	0.862	0.422	14.075 ± 6.802	0.166	0.847
	Hui(1188)	7.285 ± 3.406			6.739 ± 3.216			14.024 ± 6.228		
	others(15)	7.533 ± 3.681			5.600 ± 2.823			13.133 ± 5.680		
Only Child	Yes(363)	7.305 ± 3.650	0.001	0.999	6.907 ± 3.592	1.179	0.239	14.185 ± 6.862	0.612	0.541
	No(2150)	7.305 ± 3.521			6.695 ± 3.345			13.992 ± 6.468		
Feelings of family SES	Yes(1768)	7.515 ± 3.432	4.425^**^	0.000	6.932 ± 3.305	4.281^**^	0.000	14.447 ± 6.323	4.597^**^	0.000
	No(745)	6.809 ± 3.737			6.285 ± 3.527			13.094 ± 6.905		
Parental marriage	Normal(2145)	7.247 ± 3.491	2.016	0.133	6.685 ± 3.347	2.147	0.117	13.932 ± 6.432	2.245	0.106
	Remarried(125)	7.608 ± 3.600			7.224 ± 3.569			14.832 ± 6.836		
	single parent(243)	7.666 ± 3.896			7.666 ± 3.896			14.642 ± 7.152		
Father education	Primary school (2016)	7.350 ± 3.511	1.272	0.203	6.764 ± 3.370	0.723	0.470	14.115 ± 6.497	1.064	0.287
	Middle school or above(497)	7.125 ± 3.650			6.642 ± 3.446			13.767 ± 6.655		
Mother education	Primary school (1316)	7.505 ± 3.378	2.947^**^	0.003	6.858 ± 3.281	1.824	0.068	14.363 ± 6.279	2.543^*^	0.011
	Middle school or above(1197)	7.087 ± 3.697			6.611 ± 3.493			13.698 ± 6.779		
Worries about COVID-19	little(665)	7.015 ± 3.721	3.206*	0.041	6.731 ± 3.625	0.071	0.931	13.746 ± 7.011	0.904	0.405
	somewhat(1090)	7.360 ± 3.560			6.767 ± 3.389			14.127 ± 6.519		
	more(758)	7.483 ± 3.329			6.710 ± 3.157			14.193 ± 6.091		

^**^P < 0.01, ^*^P < 0.05

The descriptive statistics and correlation analysis of the variables are shown in Table 2. Self-esteem, resilience were negatively correlated with social distress scores (*r* = -0.411, *p* < 0.01; *r* =-0.387, *p* < 0.01,respectively). Self-esteem and resilience were negatively correlated with social avoidance scores (*r* = -0.437, *p* < 0.01; *r* = -0.379, *p* < 0.01, respectively). Social distress and social avoidance scores were positively correlated (*r* = 0.778, *p* < 0.01).

**Table 2:** Mean, standard deviation and correlation (r) of variables

Variables	$\bar X$ ± s	1	2	2.1	2.2	2.3	3	4
1. self-esteem	26.640 ± 3.701	1						
2. resilience	57.663 ± 20.095	0.378^**^	1					
2.1 optimism	8.493 ± 3.385	0.266^**^	0.850^**^	1				
2.2 strength	19.427 ± 6.672	0.382^**^	0.964^**^	0.797^**^	1			
2.3 tenacity	29.742 ± 11.030	0.376^**^	0.978^**^	0.760^**^	0.906^**^	1		
3. social distress	7.305 ± 3.539	-0.411^**^	-0.387^**^	-0.300^**^	-0.359^**^	-0.397^**^	1	
4. social avoidance	6.740 ± 3.384	-0.437^**^	-0.379^**^	-0.289^**^	-0.359^**^	-0.385^**^	0.778^**^	1

^**^P < 0.01

Table 3 presents the results of the hierarchical multiple linear regression analysis of the factors associated with social avoidance in students. The self-esteem in Model 2 were significantly negatively correlated with social avoidance (β = -0.394, *p* < 0.01). Moreover, in model 3, resilience (β = -0.038, *p* < 0.01) was significantly negatively associated with social avoidance. In model 4, social distress (β = 0.669, *p* < 0.01) was significantly positively associated with social avoidance. Model 2(R^2^ = 0.219, ∆R^2^ = 0.182) was better than model 1 in explaining social avoidance, and model 3(R^2^ = 0.261, ∆R^2^ = 0.042) was better than model 2, then model 4(R^2^ = 0.627, ∆R^2^ = 0.368) was better than model 3.

**Table 3:** The factors associated with social avoidance: hierarchical multiple linear regression

Variables	Model1	Model2	Model3	Model4
	β	β	β	β
Gender	1.049^**^	1.029^**^	0.728^**^	0.246^**^
Residence	-0.363^*^	-0.341^*^	-0.361^**^	-0.145
Ethnic	-0.080	-0.094	-0.122	-0.018
Only Child	-0.386	-0.238	-0.175	-0.174
Feelings of family SES	-0.752^**^	-0.411^**^	-0.386^**^	-0.2162
Parental marriage	0.084	0.037	0.054	-0.042
Father education	0.020	0.109	0.105	0.042
Mother education	-0.313^*^	-0.192	-0.189	0.019
Worried about COVID-19	-0.123	-0.060	-0.097	-0.182^**^
Self-esteem		-0.394^**^	-0.316^**^	-0.116^**^
Resilience			-0.038^**^	-0.009^**^
Social distress				0.669^**^
Constant	8.160^**^	17.587^**^	18.133^**^	6.242^**^
F	10.641^**^	70.111^**^	80.148^**^	352.718^**^
R^2^	0.037	0.219	0.261	0.627
∆R^2^	0.037	0.182	0.042	0.368

^**^p < 0.01, ^*^p < 0.05

The results of above analyses meet the statistical requirements for further intermediate validity testing of resilience and social distress [45]. After setting gender as control variable, the mediating effects of resilience and social stress on the relationship between self-esteem and social avoidance among college students were tested. The results showed that the mediation model of self-esteem and social avoidance had good fit indices [46]: χ^2^/df = 10.02, CFI = 0.993, TLI = 0.983, RMSEA = 0.060, SRMR = 0.015.

The regression results are shown in Table 4: Self-esteem of college students can significantly negatively predict social avoidance(β=-0.117, *p* < 0.001) and social distress(β=-0.303, *p* < 0.001), significantly positively predict resilience (β = 0.292, *p* < 0.001); resilience can significantly and negatively predict social distress(β=-0.307, *p* < 0.001) and avoidance (β=-0.067, *p* < 0.001); social distress can significantly and positively predict social avoidance(β = 0.669, *p* < 0.001).

**Table 4:** A test of the direct effects of self-esteem, resilience, social distress and social avoidance

Outcome variable	Predictor variable	β	Se	t	LLCI	ULCI
Resilience	self-esteem	0.292	0.014	21.395^**^	0.265	0.319
Social distress	self-esteem	-0.303	0.02	-15.214^**^	-0.342	-0.264
social distress	Resilience	-0.307	0.026	-11.79^**^	-0.358	-0.256
social avoid	self-esteem	-0.117	0.014	-8.171^**^	-0.145	-0.089
social avoid	Resilience	-0.067	0.015	-4.512^**^	-0.097	-0.038
social avoid	Social distress	0.669	0.014	47.873^**^	0.642	0.697

^**^p < 0.001

**Fig. 1:**
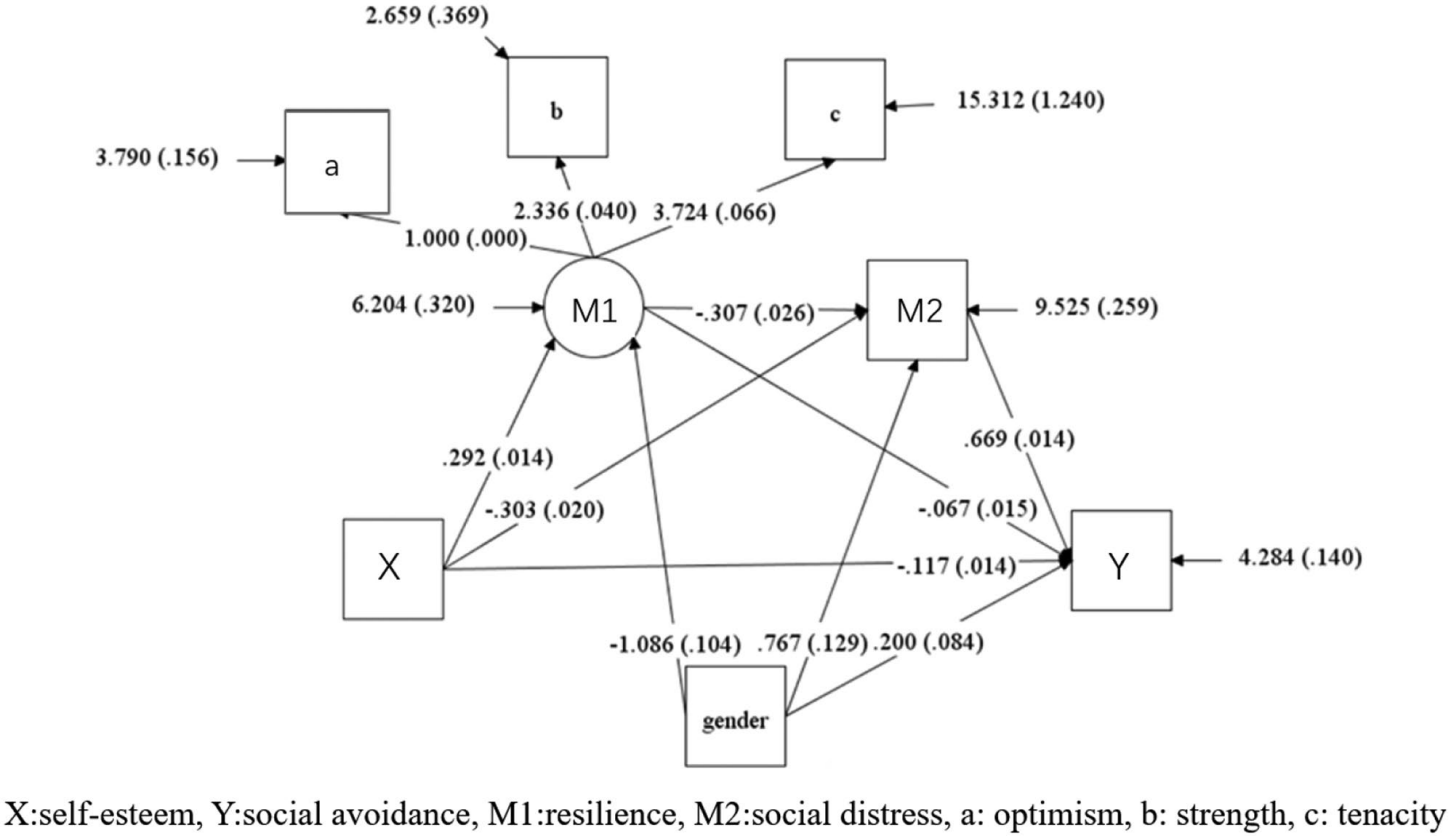
Mediating effect model of self-esteem, resilience, social distress, and avoidance

After further conducting mediation effect analysis, the results are shown in Table 5; Fig. 1. The direct effect of self-esteem on social avoidance among college students is -0.117, and the confidence interval does not include 0, indicating that the direct effect is significant, accounting for 29.32% of the total effect; the total indirect effect value is -0.282, and the confidence interval does not include 0, indicating that the mediating effect of resilience and social distress between college students’ self-esteem and social avoidance is significant, accounting for 70.67% of the total effect. Among them, the path effect value using resilience as the mediating variable is -0.02, 95%CI=[-0.028, -0.011]; the path effect value using social distress as the mediating variable is -0.203, 95%CI=[-0.229, -0.176]; while using resilience and social distress as mediating variables, the chain mediation path effect value is -0.06, 95%CI=[-0.07, -0.05]. It can be seen that the above three indirect effect paths are all significant.

**Table 5:** Path coefficients among structural equation modeling

Path	Effect size	Boot SE	LLCI	ULCI	Percentage of Total Effect
Self-esteem (X) → Resilience(M1) →Avoidance(Y)					
Self-esteem (X) → Distress(M2) →Avoidance(Y)					
Self-esteem (X) →Resilience (M1) → Distress(M2)→Avoidance(Y)					
Total	-0.399	0.019	-0.436	-0.363	
Direct effect (X) →(Y)	-0.117	0.014	-0.145	-0.089	29.32%
Total indirect effect	-0.282	0.014	-0.309	-0.255	70.67%
(X) → (M1) →(Y)	-0.02	0.004	-0.028	-0.011	5.01%
(X) → (M2) →(Y)	-0.203	0.014	-0.229	-0.176	50.87%
(X)→(M1) →(M2)→(Y)	-0.06	0.005	-0.07	-0.05	15.03%

X: self-esteem, Y:social avoidance, M1:resilience, M2:social distress, a: optimism, b: strength, c: tenacity

## Discussion

This study used college students as the research subjects and investigated the relationships between social avoidance and self-esteem, resilience and social distress, as well as the underlying mechanisms. Descriptive analysis revealed differences in social distress/avoidance among demographic data such as gender, feelings of family SES, mother’s education level, and worries about COVID-19, while the rest of the data showed no differences. Females’ social distress/avoidance is higher than males’, probably due to the fact that girls are delicate-minded and pay more attention to other people’s evaluation and opinion of themselves in social situations, which tends to produce excessive distress and worry in the process of socialising; they are prone to negative manifestations of suspicion, anxiety, and sentimentality in dealing with interpersonal relationships, and they deal with them in a relatively restrained and implicit manner, which easily leads to anxiety/avoidance [47, 48], which is more in line with the Chinese culture of women’s characteristics of women. Students who do not feel stress of family SES are more confident, have more resources, and are more at ease in interpersonal interactions, which helps alleviate social distress/avoidance [49]. In life, mothers provide more care for their children, and parents with a certain level of education adopt more tolerant and wise approaches to educating them. They can offer timely and effective assistance when their children encounter setbacks, helping them learn proper interpersonal skills and alleviate social anxiety [50]. In the context of the COVID-19 pandemic, college students did indeed experience an increase in social distress in their interactions [51, 52].

The current study offers evidence that low self-esteem is associated with a higher risk of social avoidance, and Hypothesis 1 is validated. Consistent with most previous research, Calin [53] overviewing the relationship between self-esteem and social anxiety showed that low self-esteem could increase the likelihood of social anxiety, including social distress and avoidance. He et al. [54] found that people with low self-esteem have a lower sense of self-identity, a more negative self-perception, and tend to be overly concerned about negative evaluations from others in interpersonal interactions, leading to distress and avoidance from excessive worry during social interaction. It means that those with high self-esteem have lower levels of social anxiety and less social avoidance [34, 53, 55, 56]. Individuals with higher self-esteem have greater social self-efficacy, effectively regulating themselves. They can handle social issues calmly and proactively in social situations [57, 58], and thus experience less social distress and avoidance. We also found that social distress partially mediates the association between self-esteem and avoidance, with a mediating effect of 50.87%, and tested Hypothesis 3. According to ABC theory, social anxiety and avoidance behaviors are associated with negative cognition, excessive worry, and fear of negative evaluations when interacting with others. Low self-esteem individuals have a lower sense of self-identity and value, tend to be more negative about themselves, focus excessively on others’ negative evaluations of them in interpersonal interactions [59], and are more likely to reflect on their flaws rather than achievements [60, 61]. They might think that they are not good enough to interact with others, leading to an increase in social distress/avoidance. By improving their self-esteem, they can better accept themselves, manage others’ negative evaluations in social activities, reduce social distress [57], and thereby reduce social avoidance behaviours. Therefore, promoting students’ self-esteem is crucial to reducing social avoidance, and improving negative perceptions in social interactions can also help to reduce social distress in students with low self-esteem.

The analysis in the paper demonstrates that resilience is not only negatively associated with social avoidance, but also partially mediates the association between self-esteem and avoidance. Hypothesis 2 was tested. Resilience is how well an individual adapts to the life adversity, trauma, tragedy, threats, or other major stressors in life, and it implies the ability to bounce back in the face of life’s stresses and setbacks [62]. Compared to students with high resilience, students with low resilience are more vulnerable and sensitive [63]. It is difficult for them to fully mobilise positive psychological resources to cope with complex interpersonal relationships, leading to intense interpersonal relationships, which is the direct cause of social anxiety [64]. Resilience, as an individual’s positive psychological trait, has the ability to reorganize and transform external risk factors into protective ones, helping people find reliable social support, stay optimistic, and experience positive emotions [65]; it can successfully overcome negative cognition, obstacles, alleviate anxiety, and avoidance behaviors. This study found that resilience mediates the relationship between self-esteem and social avoidance by 5%. Specifically, even students with low self-esteem can actively use resources, manage stress, and learn from stress experiences to better cope with social problems and reduce barriers to living and learning.

### Chain mediating role of resilience and distress

The present study showed that self-esteem influences social avoidance among university students through the chain mediating effect of resilience and social distress, and the hypothesis 4 was tested. This study suggests that higher resilience among university students is associated with lower social distress, consistent with most previous research [37, 66]. Studies have shown that higher resilient adolescents cope with psychological distress more effectively because they are more likely to adopt a positive attitude and optimistic beliefs when facing life-threatening situations, and higher resilient youth often have positive personal traits, including good life managing skills, self-efficacy, and perceived competence [37]. Therefore, despite exposure to stressors, resilience can buffer the negative effects of emotional distress, buffer the psychological pressure caused by life stress, maintain health levels, and improve interpersonal relationships [67]. Some researches suggested that low levels of resilience may also buffer individuals from low to moderate levels of anxiety symptoms in stressful situations [22, 68]. The value-added spiral effect in the theory of resource conservation suggests that an individual with a positive resource not only has the ability to acquire other positive resources, but that the acquired resources produce a greater incremental increase in resources. Self-esteem and psychological resilience are seen as positive psychological resources for individuals [19]. Self-esteem and resilience can add value to resources by acting synergistically and cumulatively to diminish resources that impede an individual’s development and reduce an individual’s level of social distress, which can enhance an individual’s positive psychological state overall and drive college students to exhibit less social avoidance. Therefore, by maintaining college students’ good self-esteem, increasing their resilience and helping them to access positive psychological resources, their social distress can be reduced and social avoidance can be alleviated.

### Limitations and prospects

This study has several main limitations. First, the study sample was confined to university students from a single school in Ningxia, which limits the generalizability of the findings. In future studies, there may be a need for sampling in more areas in order to enrich the sample and reduce its limitations. Second, the study is cross-sectional data, it lacks temporal evidence for causality. Future studies should use longitudinal designs to explore the causal relationships between different variables. Third, the data came from subjective self-rating reports, which may result in deviations. Although there was no common method deviation after data verification, the results still may not completely match the actual situation due to the influence of social approval and other factors. In order to improve the reliability and validity of studies, multiple methods can be used to collect data simultaneously in the future. Finally, there may be mechanisms involving other variables between self-esteem and social avoidance. The emergence of a behaviour is related to individual (e.g. mental health), environmental and social factors. In this study, the changes in the coefficients of the demographic factors identified in the regression analyses as the variables increase suggest that there is an interaction between the variables and this is an area for future research. It is also worth exploring a more nuanced categorisation of the variables, e.g. too low or too high self-esteem can be detrimental [69] and there is a need to further differentiate between these two states in relation to the variables. Clarifying the internal mechanisms of social avoidance will help to develop better intervention strategies.

## Conclusions

The current study explores the associations between self-esteem and social avoidance among students in Ningxia of China, and examines the role of resilience and social distress in addressing social avoidance. We found that self-esteem is negatively associated with social avoidance. Furthermore, resilience and social distress hold promise as meaningful factors that can partially mediate the associations. These findings would suggest that interventions targeting resilience and social distress may be a preventive measure against social avoidance in low self-esteem students. In this respect, by strengthening home-school links, parents and teachers should encourage students to enhance students’ self-esteem and foster resilience to help students positively cope with social stresses and challenges. Additionally, school-based educators and relevant authorities should conduct regular mental health monitoring and evaluation, which can identify social anxiety levels promptly to mitigate the onset of social avoidance.

The results of the study provide important information that can be applied to related theories and preventive interventions against social avoidance in college students. In theory, this study proposed that self-esteem effects the level of avoidance through multiple pathways, and identified two important factors mediating the relationship between self-esteem and avoidance in college students: resilience and social distress. Research shows that we should pay more attention and support to students who avoid social interaction. We should try our best to increase college students’ self-esteem and resilience in order to reduce the possibility of social distress and avoidance. In practice, the results of this study make vital sense to the interventions and prevention of college students’ social anxiety. It is suggested that psychology experts should regularly organize special lectures or set up training courses on resilience and social distress adjustment skills in colleges and universities to improve the level of resilience of students. In addition, schools provide corresponding group counseling activities with the theme of psychological resilience to help students accept themselves and each other through social skills training, role-playing, and joint discussions. This will help them master communication skills and increase their communication-related confidence and self-esteem, so that they can obtain social support through communication with others in a group atmosphere. Furthermore, individual counselling should be offered to those students who show significant social avoidance, low self-esteem or low psychological resilience. Additionally, if the above methods prove ineffective, school counsellors have the responsibility to recommend that students with more severe social anxiety be referred to a psychologist or psychiatrist. In conclusion, early and effective intervention at multiple levels for college students with social anxiety can enable them to cope with life rationally and well, thus promoting their physical and mental health.

## Data Availability

Data is provided within the manuscript or supplementary information files.

## Abbreviations

SA: Social Anxiety
SAD: Social Avoidance and Distress Scale
RSES: Rosenberg Self-Esteem Scale
CD: RISC-Connor-Davidson Resilience Scale
SES: Socioeconomic status
CFI: Comparative Fit Index
TLI: Tucker-Lewis Index
RMSEA: Root Mean Square Error of Approximation
SRMR: Standardized Root Mean Square Residual
LLCI: Lower Level of Confidence Interval
ULCI: Upper Level of Confidence Interval
Boot SE: Bootstrap standard error
